# Immunoglobulin Light Chain Amyloidosis

**DOI:** 10.1002/ccr3.72958

**Published:** 2026-06-16

**Authors:** Justin Lyon, Ashley Khouri, Katheryn A. Bell, Jamie Zussman

**Affiliations:** ^1^ Spencer Fox Eccles School of Medicine Salt Lake City Utah USA; ^2^ Department of Dermatology University of Utah Salt Lake City Utah USA

**Keywords:** amyloidosis, cutaneous, macroglossia, multiple myeloma, nodule, plaque

## Abstract

Symmetric, purpuric hand nodules and macroglossia prompted skin biopsy revealing immunoglobulin light chain amyloidosis, leading to the diagnosis of multiple myeloma. Early detection of typical and atypical cutaneous amyloidosis presentations, such as this patient, is imperative for clinicians to initiate timely hematologic workup and treatment to improve patient outcomes and survival.

## Graphical abstract

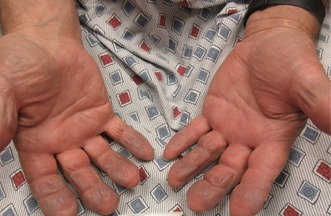

## 1 Introduction

Immunoglobulin light chain amyloidosis is a plasma cell disorder defined by the deposition of misfolded monoclonal light chains into organ systems including the lungs, kidney, skin, and heart [1]. Clinical features are largely dependent upon the involved tissues but may include conditions such as heart failure with preserved ejection fraction (HFpEF), nephrotic syndrome, hepatic dysfunction, peripheral neuropathies as well as cutaneous manifestations [2]. Skin findings may include periorbital ecchymoses, easy bruising, nail dystrophy, macroglossia as well as the presence of yellowish waxy, smooth papules and nodules distributed along the periorificial, flexural or anogenital regions [3, 4]. Herein we describe a case of immunoglobulin light chain amyloidosis with unique cutaneous manifestations that led to a diagnosis of multiple myeloma, and illustrate the importance of early recognition for improved long‐term survival and symptom control.

## 2 Case History/Examination

**FIGURE 1:**
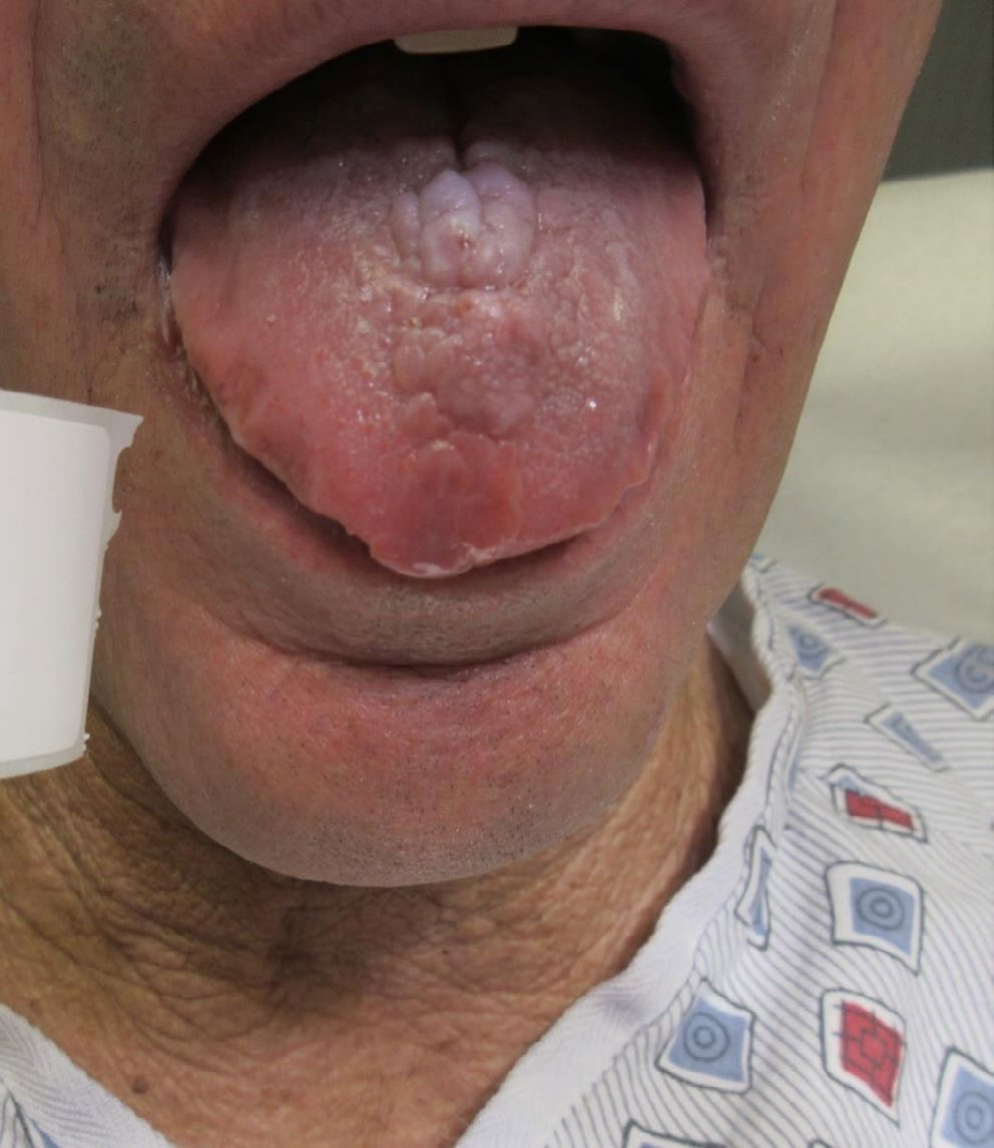
Macroglossia with lateral scalloping.

**FIGURE 2:**
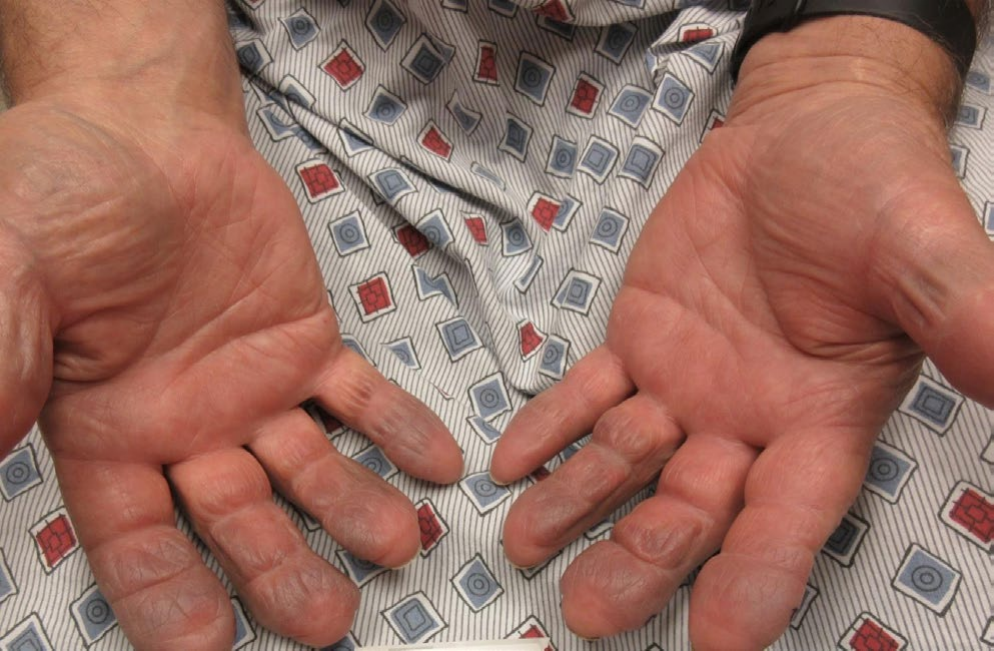
Waxy, purple nodules and plaques distributed across the distal fingers and thenar eminences.

A 78‐year‐old male with a past medical history of hypertension and hyperlipidemia presented to the Veterans Affairs dermatology clinic as a new patient for evaluation of noticeable enlargement of the hands appearing approximately two years prior. He also reported significant fatigue, as well as recent onset of joint pains in his back and extremities. Upon further questioning, the patient also noted some distortion and enlargement of his tongue in the months prior to presentation. Examination of the patient's mouth was notable for an enlarged tongue with lateral scalloping and ventral nodularity with white‐purple and red discoloration (Figure 1). The patient's hands were thickened and enlarged with ill‐defined, purple, doughy‐feeling plaques spanning the distal digits on the volar surface bilaterally and symmetrically from the proximal inter‐phalangeal joints to the tips of the fingers, as well as the thenar eminences (Figure 2).

## 3 Differential Diagnosis, Investigations, and Treatment

**FIGURE 3:**
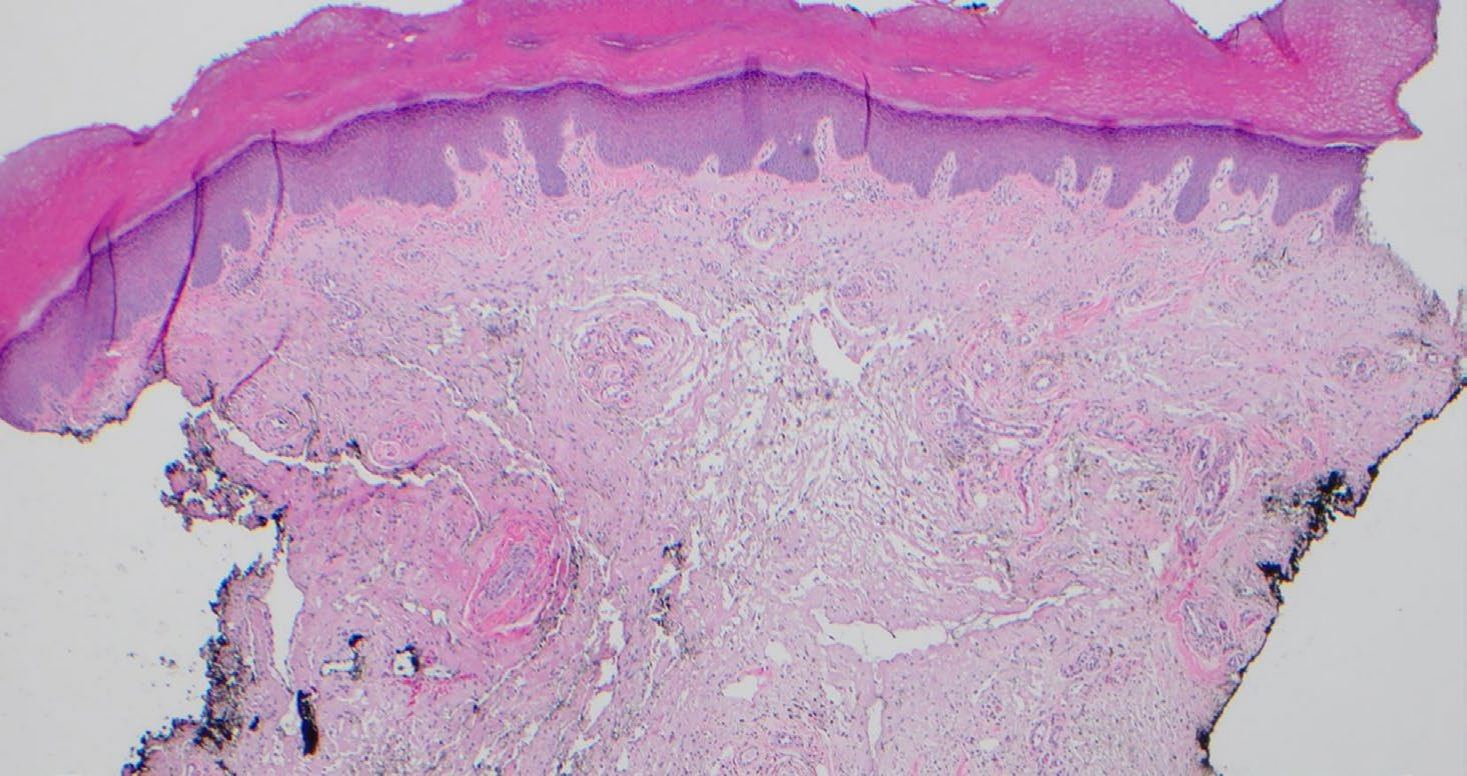
Hematoxylin and eosin staining demonstrates dermal deposits of amorphous eosinophilic material with a sparse lymphoplasmacytic infiltrate. 100×.

**FIGURE 4:**
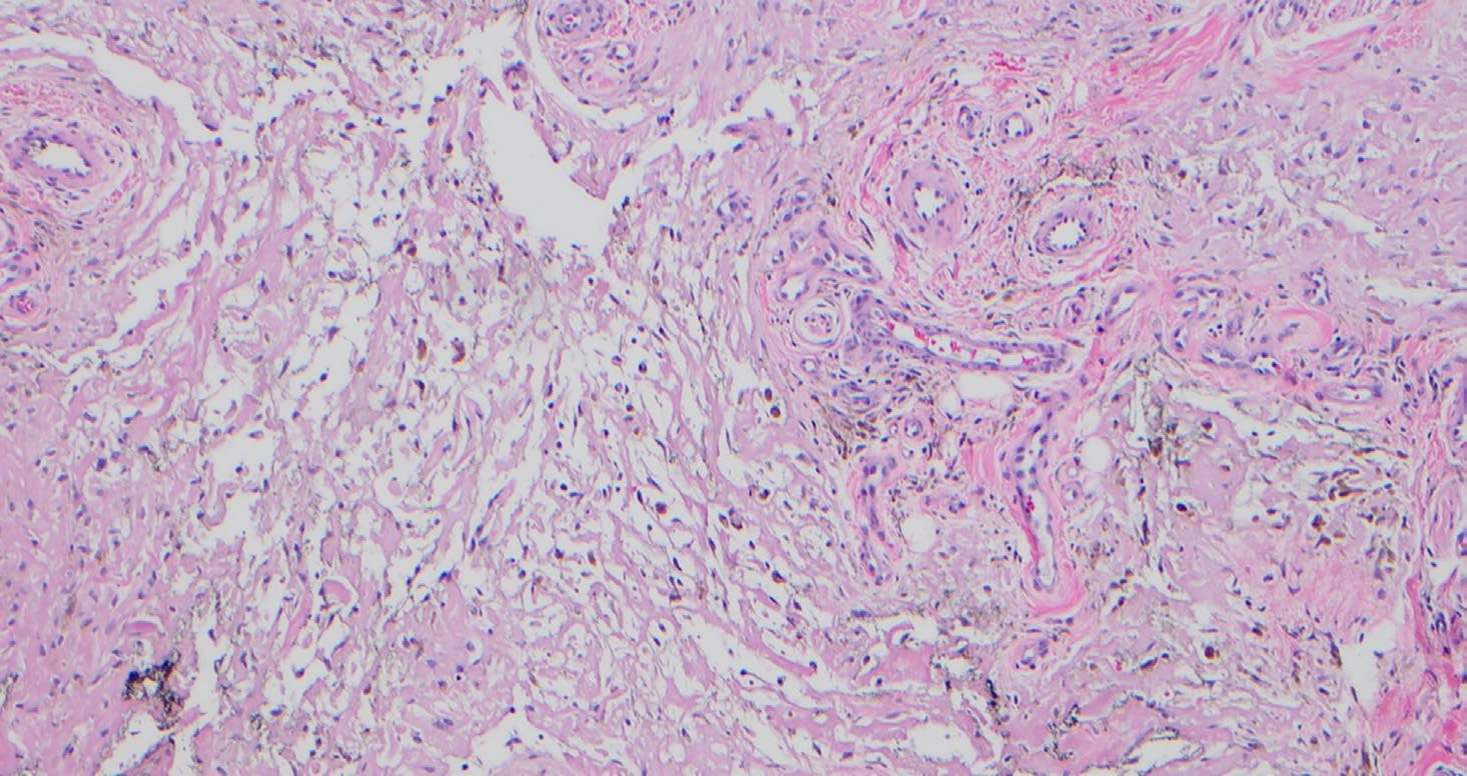
Higher power view of the amorphous eosinophilic material consistent with amyloid. 400×.

**FIGURE 5:**
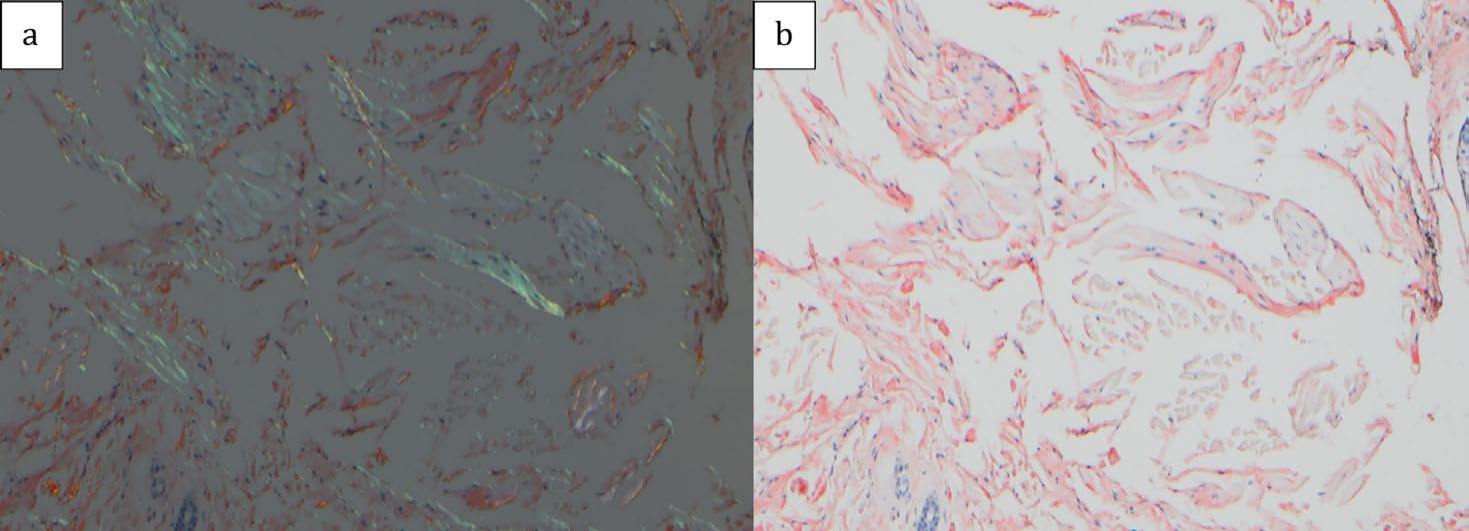
(a) Congo red staining highlights the amyloid deposits (b) Using polarized light the amyloid deposits demonstrate classic apple‐green birefringence. 100×.

A punch biopsy of the left first and second phalange was performed showing eosinophilic amorphous material in the dermis, associated with a mild lymphoplasmacytic perivascular infiltrate, changes consistent with amyloidosis (Figures 3 and 4). Congo red staining demonstrated apple‐green birefringence under polarized light, confirming the presence of amyloid deposits (Figure 5). Initial laboratory studies uncovered a monoclonal gammopathy with an increase in lambda free light chains (1318.96, ref range 5.71–26.30) and a kappa to lambda free light chain ratio of 0.02 (ref range 0.26–1.65). The patient was referred to hematology/oncology where a bone marrow biopsy with flow cytometry confirmed a diagnosis of multiple myeloma, demonstrating 30% clonal plasma cells. Among the CRAB criteria, the patient demonstrated renal impairment (elevated creatinine), but lacked hypercalcemia, anemia, or lytic bone lesions on PET/CT imaging.

## 4 Conclusions and Results

This presentation was distinguished from MGUS‐associated AL by the degree of clonal plasma cell expansion on bone marrow biopsy. Dara‐CyBorD therapy was initiated and after months of therapy, notable improvement in symptoms along with normalization of his serum free light chains was observed. Following treatment, his creatinine improved initially before stabilizing; he carries an established diagnosis of chronic kidney disease stage III attributed to amyloidosis. His HFpEF has remained stable with preserved ejection fraction and stable echocardiographic findings on follow‐up. A repeat bone marrow biopsy showed no evidence of residual plasma cell dyscrasia. He is now continuing monthly maintenance with Daratumumab.

## 5 Discussion

We present a case of immunoglobulin light chain (AL) amyloidosis manifesting in the skin secondary to underlying multiple myeloma. Common cutaneous manifestations of AL amyloidosis include macroglossia with lateral scalloping, purpura, petechiae, and ecchymoses (commonly in a peri‐orbital distribution), bullous lesions, nodular lesions, scleroderma‐like changes, and alopecia [3, 4]. Though our patient presented with the classic symptom of macroglossia, this was not something he initially reported to us, and he lacked classic peri‐orbital lesions or widespread ecchymoses. It is worth noting that periorbital ecchymoses and macroglossia, while considered canonical features of AL amyloidosis, are present in only a minority of patients, typically those with more advanced disease, and their absence should not exclude the diagnosis. The symmetric disfiguration of his hands with purpuric nodules was a unique presentation not previously described, and something that had gone unrecognized by his previous non‐dermatologic providers for over two years—during which time he had undergone an extensive work‐up for fatigue and joint pain. The violaceous discoloration of the nodules in his hands was a helpful clue to diagnosis, a clear clinical consequence of amyloid deposition within vessel walls causing fragility and red cell extravasation.

Immunoglobulin light chain (AL) amyloidosis is a rare condition with a current annual incidence of 16.7 per million person‐years and a point prevalence of 69.0 per million adults [5]. These statistics represent a trend toward increasing prevalence; however, this is likely related to prolonged survival rather than a true rise in cases per year. Demographically, AL amyloidosis is more common in older adults, with a median age at time of diagnosis of just over 60. The condition has a slight male predominance, and patients often present for evaluation due to symptoms in organ systems secondarily affected (kidneys, skin, heart) [5]. Organ involvement is a key prognostic factor in addressing patient risk stratification and care management. This condition arises almost exclusively in the context of clonal plasma cell disorders, most notably monoclonal gammopathy of undetermined significance (MGUS) or multiple myeloma, as seen in this patient [5, 6, 7].

Skin biopsy of a nodular cutaneous lesion demonstrates extensive amyloid deposits in the dermis, subcutis, and around blood vessels [2]. This allows histologic distinction from primary cutaneous lichen and macular amyloidosis which demonstrate keratin‐derived amyloid localized to the papillary dermis. Any patient found to have nodular deposits of amyloid in the skin should be evaluated for a monoclonal plasma cell proliferative disorder [2]. Initial screening may be performed with serum protein electrophoresis and/or serum immunofixation electrophoresis. If initial results suggest a plasma cell dyscrasia, additional studies may include cardiac biomarkers, echocardiography, as well as bone marrow biopsies [6, 7]. In the case of our patient, further workup identified heart failure with preserved ejection fraction (HFpEF) secondary to amyloid deposition along with a history of neuropathic symptoms. Kidney involvement may be seen in patients with plasma cell dyscrasias, however, no confirmatory biopsy was performed to confirm the presence of amyloid infiltrative disease.

This case is yet another clear example of how recognizing the cutaneous manifestations of internal disease, even when the presentation is not classic, can lead to proper diagnosis and targeted care for our patients, ultimately improving their quality of life.

## Author Contributions

**Justin Lyon:** conceptualization, investigation, methodology, writing – original draft, writing – review and editing. **Ashley Khouri:** writing – review and editing. **Katheryn A. Bell:** conceptualization, methodology, resources, supervision, validation, writing – review and editing. **Jamie Zussman:** conceptualization, formal analysis, investigation, methodology, project administration, supervision, validation, visualization, writing – review and editing.

## Funding

The authors have nothing to report.

## Ethics Statement

The authors have nothing to report.

## Consent

Written patient consent was obtained prior to completion of this manuscript.

## Conflicts of Interest

The authors declare no conflicts of interest.

## Data Availability

Data sharing not applicable to this article as no datasets were generated or analyzed during the current study.
